# Annotation of 2,507 *Saccharomyces cerevisiae* genomes

**DOI:** 10.1128/spectrum.03582-23

**Published:** 2024-03-15

**Authors:** Meng Wang, Xuan Li, Xian Liu, Xiaoping Hou, Yang He, Jun-Hong Yu, Shumin Hu, Hua Yin, Bin-Bin Xie

**Affiliations:** 1 Microbial Technology Institute and State Key Laboratory of Microbial Technology, Shandong University, Qingdao, China; 2 State Key Laboratory of Biological Fermentation Engineering of Beer, Tsingtao Brewery Co., Ltd, Qingdao, China; University at Buffalo, State University of New York, Buffalo, New York, USA

**Keywords:** *Saccharomyces cerevisiae*, genome, annotation

## Abstract

*Saccharomyces cerevisiae* (baker’s yeast, budding yeast) is one of the most important model organisms for biological research and is a crucial microorganism in industry. Currently, a huge number of *Saccharomyces cerevisiae* genome sequences are available at the public domain. However, these genomes are distributed at different websites and a large number of them are released without annotation information. To provide one complete annotated genome data resource, we collected 2,507 *Saccharomyces cerevisiae* genome assemblies and re-annotated 2,506 assemblies using a custom annotation pipeline, producing a total of 15,407,164 protein-coding gene models. With a custom pipeline, all these gene sequences were clustered into families. A total of 1,506 single-copy genes were selected as marker genes, which were then used to evaluate the genome completeness and base qualities of all assemblies. Pangenomic analyses were performed based on a selected subset of 847 medium-high-quality genomes. Statistical comparisons revealed a number of gene families showing copy number variations among different organism sources. To the authors’ knowledge, this study represents the largest genome annotation project of *S. cerevisiae* so far, providing rich genomic resources for the future studies of the model organism *S. cerevisiae* and its relatives.

**IMPORTANCE:**

*Saccharomyces cerevisiae* (baker’s yeast, budding yeast) is one of the most important model organisms for biological research and is a crucial microorganism in industry. Though a huge number of *Saccharomyces cerevisiae* genome sequences are available at the public domain, these genomes are distributed at different websites and most are released without annotation, hindering the efficient reuse of these genome resources. Here, we collected 2,507 genomes for *Saccharomyces cerevisiae*, performed genome annotation, and evaluated the genome qualities. All the obtained data have been deposited at public repositories and are freely accessible to the community. This study represents the largest genome annotation project of *S. cerevisiae* so far, providing one complete annotated genome data set for *S. cerevisiae*, an important workhorse for fundamental biology, biotechnology, and industry.

## INTRODUCTION

*Saccharomyces cerevisiae* (baker’s yeast, budding yeast) is not only a model organism in biological research but also has wide applications, including biotechnology, food, and pharmaceutical industries (1, 2). Since the publication of the first complete genome sequence of *S. cerevisiae* in 1996 (3), thousands of *S. cerevisiae* genome sequences have been published or released to public databases, providing rich genome information for *S. cerevisiae* studies. Recently, large-scale genome analyses revealed a pan-genome remarkably larger than the genome of the type strain S288c, which harbors approximately 6,000 genes (3). For example, comparative analysis of 1,011 genomes revealed a *S. cerevisiae* pan-genome containing 7,796 genes, including 4,940 core genes and 2,856 variable genes (4). A later study revealed 7,078 gene families from a total of 1,392 *S*. *cerevisiae* genomes (5). The large variable gene set implies genome content difference between isolates, providing opportunities to uncover the molecular basis underlying different physiology and biotechnological potentials. However, it is also noted that different studies revealed different numbers of genes in *S. cerevisiae*. Such differences would affect the comparative results of genomes from different studies, and therefore, a unified gene catalog generated from the same genome annotation pipeline will contribute to the comparative studies of *S. cerevisiae* genomes.

The *Saccharomyces* Genome Database (SGD) (https://www.yeastgenome.org/) contains rich resources for *Saccharomyces* genome studies, including annotated genomes and literature. However, only a small number of genome sequences are available in this database. The National Center for Biotechnology Information (NCBI, https://www.ncbi.nlm.nih.gov/) genome database contains over a thousand *S. cerevisiae* genomes. However, genome annotation is not available for most assemblies in the NCBI genome database. Furthermore, a large number of genomes are available as supplementary information of literature, and have not been deposited in the public databases (4). Besides, many available annotations were produced by using different bioinformatics pipelines, which might affect the results of comparative studies. All these hinder the efficient reuse of these genome resources.

**Fig 1:**
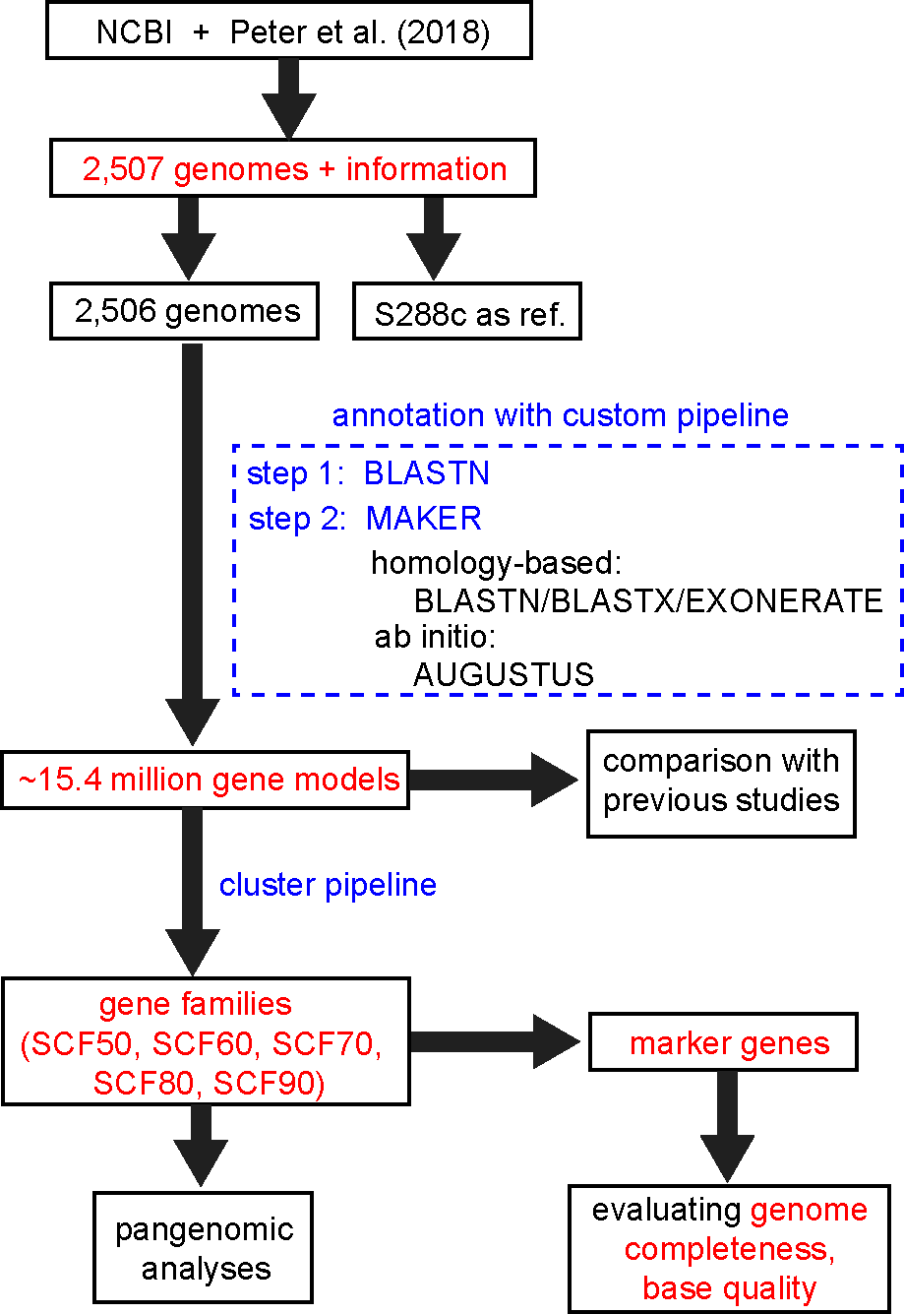
Overall flowchart of this study. Data shown in red have been deposited at figshare and Zenodo. Codes for the annotation pipeline and cluster pipeline (blue) have been deposited at GitHub.

To address the above issue, we collected 2,507 *S*. *cerevisiae* genome assemblies and re-annotated the protein-coding genes to provide uniform annotation to the community. We compiled a marker gene set specifically for *S. cerevisiae* and assessed the completeness and quality of all assemblies. We calculated the gene families based on the genome annotation. We also analyzed the *S. cerevisiae* pan-genome based on selected set of genomes. The overall flowchart of the study was shown in Fig. 1. This study provided one complete annotated genome data resource of *S. cerevisiae* genomes.

## RESULTS

### Genome data set

A total of 2,507 assemblies from two resources were obtained, including all 1,496 assemblies under the name “*Saccharomyces cerevisiae*” available from the NCBI GenBank database (December 2023) and 1,011 from a large-scale comparative genomics study of *S. cerevisiae* isolates (4). The detailed information of these genome assemblies is available in Table S1 and has also been deposited both at figshare (6) and at Zenodo (7). These assemblies showed a wide genome size range. Compared to the complete genome size 12,157,105 bp of S288c, eight assemblies had too small sizes (<10 Mb), including an assembly for the S288c mitochondrial genome (accession GCA_000091065.2, length 0.59 Mb). It was noted that 16 assemblies had large genomes (>20 Mb), including five assemblies from strains obtained through hybridization of wild and domesticated yeasts (8) and eleven phased haplotype assemblies for heterozygous diploid and polyploid isolates (9). The median size of all assemblies was 11,979,448 bp, which was slightly smaller than that of S288c (12,157,105 bp). Compared with the 16 chromosomes and one mitochondrial contig for the reference S288c genome, most assemblies were highly fragmented, with a median contig number of 1,068 and median contig N50 of 114,692 bp.

### Annotation

A two-step pipeline was used to predict protein-coding genes for all assemblies except the assembly of strain S288c (accession GCF_000146045.2), which was used as one of the reference genomes. Step 1 firstly generated models (referred to as blastn-models) at sequence regions highly similar to the reference S288c sequences (no insertions and deletions, indels, in the alignment). Then, step 2 predicted models (referred to as maker-models) at sequence regions where there were no blastn-models (sequence regions with blastn-models were masked with “N”).

**Fig 2:**
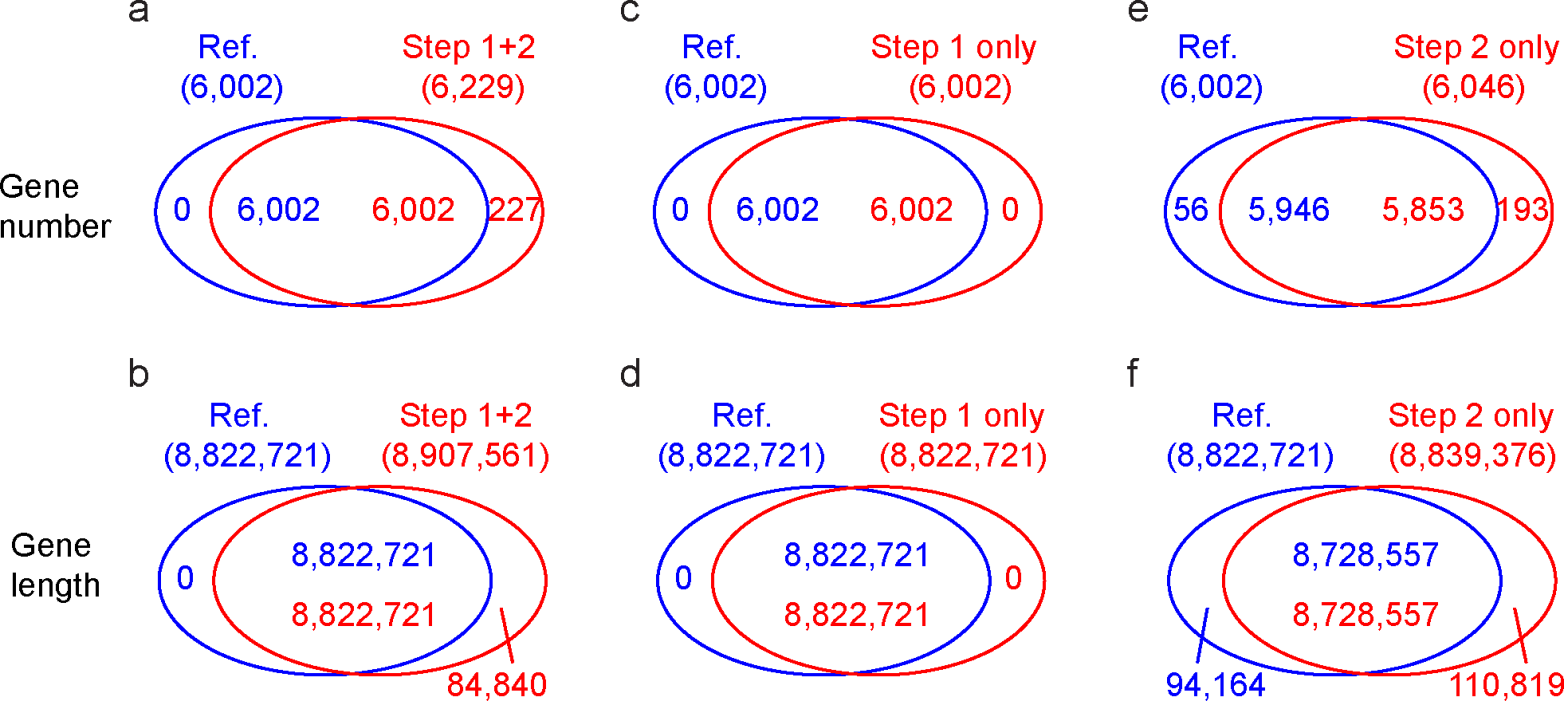
Benchmarking the genome annotation pipeline. The S288c genome has been annotated with the annotation pipeline in three modes: the complete mode (**a,b**), step 1 only mode (**c,d**), and step 2 only mode (**e,f**). The obtained models were compared with the reference models.

The S288c genome was firstly used to benchmark the pipeline (Fig. 2). The pipeline could produce 6,229 models. These included 6,002 models produced at step 1 and additional 227 models (totaling 84,840 bp) produced at step 2 (Fig. 2a and b). The 6,002 models produced at step 1 were the same as the reference models, indicating that step 1 could recover all models at sequence regions that showed high similarity to the reference S288c. Thus, the pipeline achieved a recall rate of 100% with precision of 96.4% (based on gene number) and 99.0% (based on gene length). The pipeline was also run in the step 1 only mode and the step 2 only mode, respectively. Consistent with the above results, models produced by the step 1 only mode were exactly the same as the reference models (Fig. 2c and d). The step 2 only mode recovered a total of 6,046 models, including 5,946 matching the reference models (Fig. 2e and f), and thus achieved a recall rate of 99.1% (98.9% based on gene length) and precision of 96.8% (98.7% based on gene length). These results indicated that the pipeline could recover 98.9%–99.1% of the gene models with precision of 96.8%–98.7% for genomes distantly related the S288c (high ratios of indels in the alignment). In this situation, the prediction heavily relied on step 2. In summary, the above benchmarking indicated that the pipeline developed here achieved a recall rate of 98.9%–100% and precision of 96.4%–99.0%.

**Fig 3:**
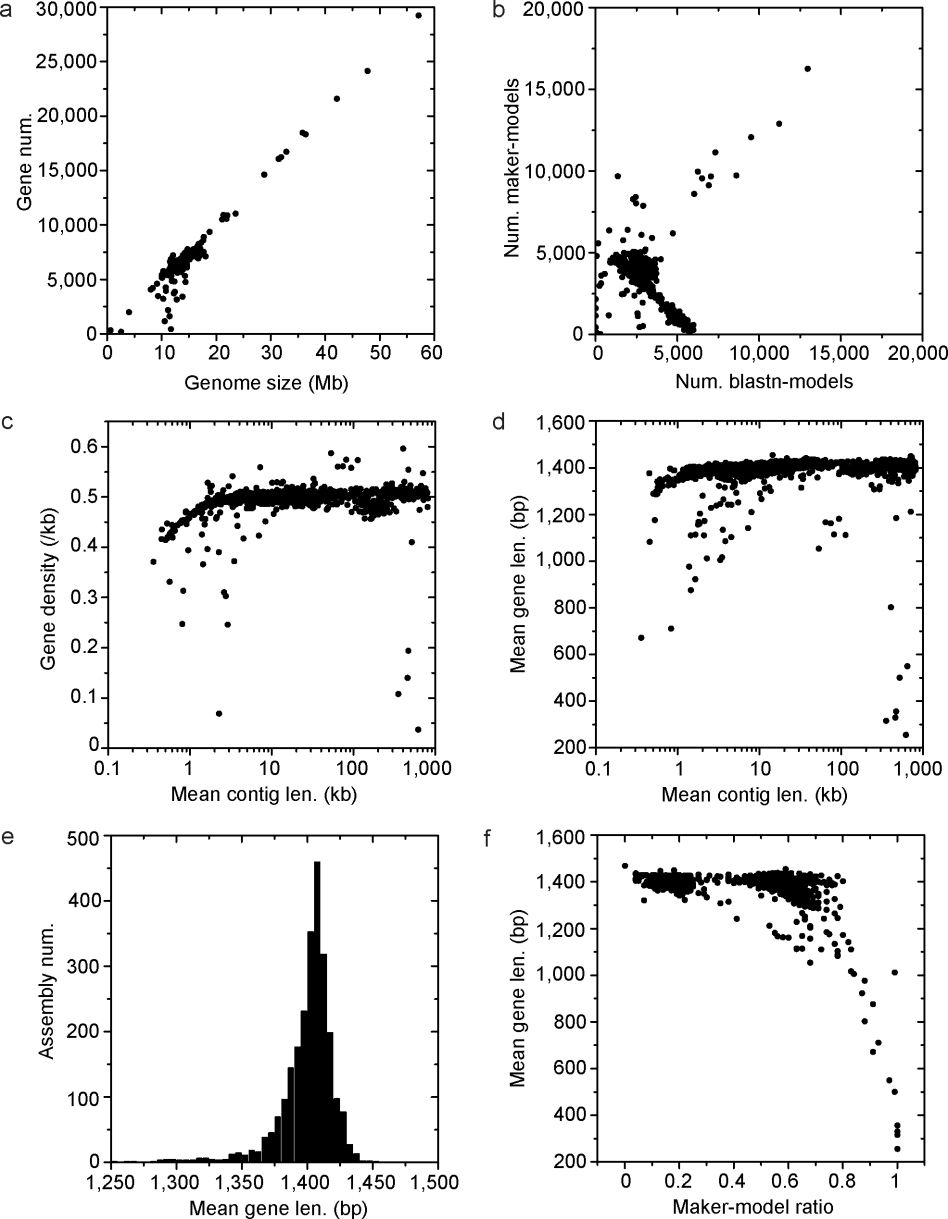
Genome sizes and gene models of the genome assemblies in this study. (a) The relationship between the gene number and the genome size. (b) The relationship between the maker-models and the blastn-models. (c) The relationship between the gene density and the mean contig length. (d) The relationship between the mean gene length and the mean contig length. (e) The distribution of the mean gene length. (f) The relationship between the mean gene length and the maker-model ratio.

Annotation with the above pipeline resulted in a final set containing 15,407,164 (including the reference S288c) gene models (6, 7, 10–17) (Fig. 3a). The median number of genes per genome (5,995) was close to that of S288c reference models (6,002). The mean gene number (6,146) was bigger than that of S288c reference models, which was probably a result of the existence of large genomes. There was a correlation between the gene number and the genome size (*r*^2^ = 0.92). Though different assemblies had various ratios of blastn-models and maker-models, the overall gene densities were similar for different assemblies (Fig. 3a and b). However, it was noted that assemblies with shorter contigs (mean contig length 1 kb) had clearly lower densities (Fig. 3c). The mean gene length of assemblies with too short contigs was also smaller than those with long contigs (Fig. 3d). This is probably a result that the pipeline failed to predict incomplete genes on the too short contigs with length close to the mean gene length (1, 468 bp for the S288c reference models and ~1.4 kb for the all assemblies studied, Fig. 3e). Besides, assemblies with too short genes had high ratios of maker-models (Fig. 3f). Our pipeline generated blastn-models on sequences (or regions) highly similar to the reference S288c genome and generated maker-models where the blastn-models were unavailable. Thus, the too low gene lengths of assemblies with too high ratios of maker-models suggested low sequence qualities of these assemblies. In addition, as shown in Fig. 3c, d, and a, few assemblies were clearly outliers with too low numbers of genes, suggesting that these genomes also had low sequence qualities, though a few of them had high continuities.

In summary, we developed and benchmarked a genome annotation pipeline and generated models for a total of 2,507 genome assemblies with this pipeline.

### Comparison with gene models obtained in other studies

**Fig 4:**
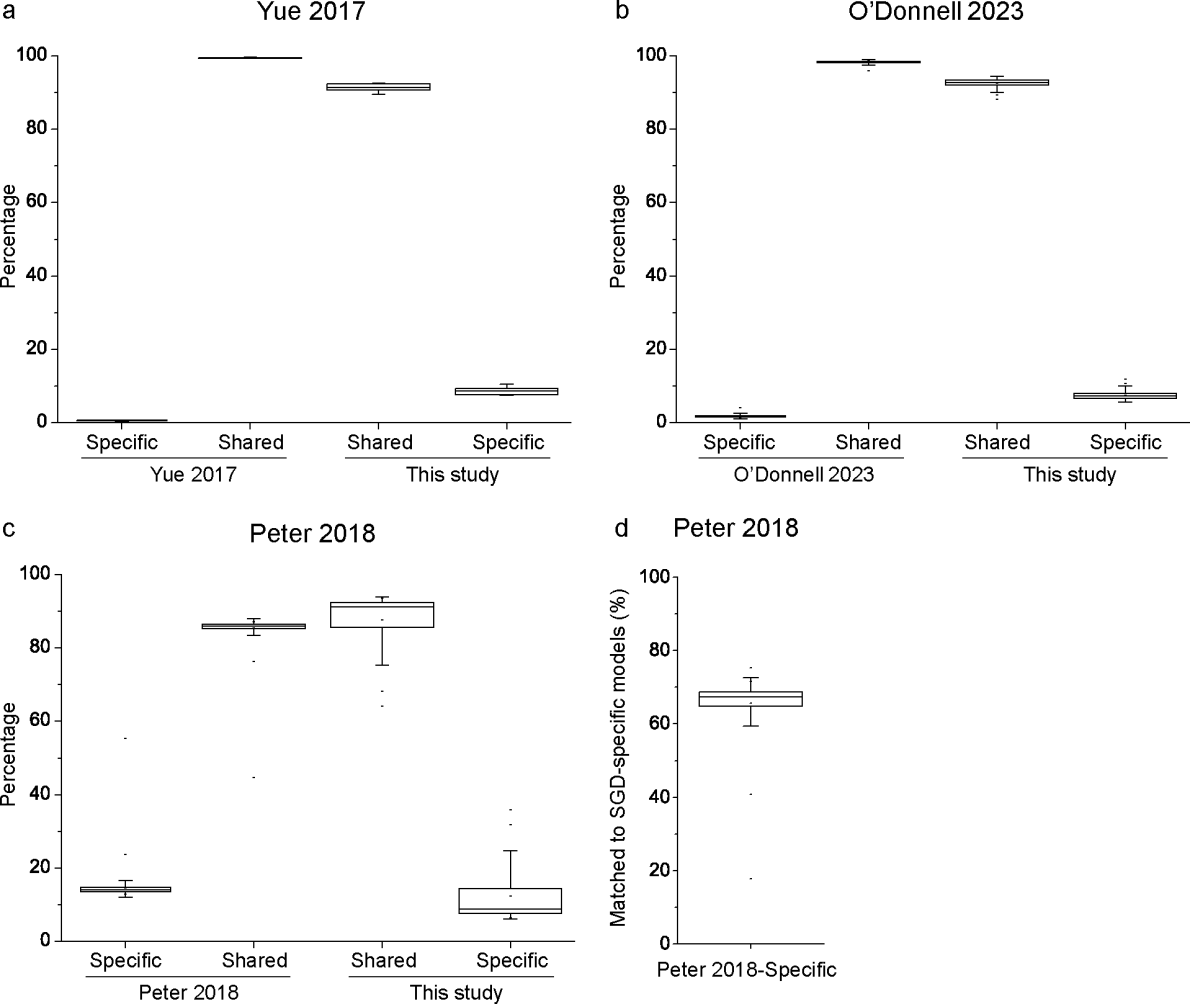
Comparison with gene models obtained in other studies.

In addition to benchmarking our pipeline with the S288c reference genome, we also compared our gene models with those obtained in other studies. Our pipeline was able to recover 99.46 ± 0.12% (mean ± standard deviation) of the protein-coding gene models in the study of Yu et al. (18) and 98.21 ± 0.59% of the protein-coding gene models in the study of O’Donnell et al. (9) (Fig. 4a and b). Furthermore, 8.64 ± 1.05% of models found by our pipeline were not reported in the study of Yu et al. (18) and 7.42 ± 1.05% of models found by our pipeline were not reported in the study of O’Donnell et al. (9).

The situation was different for the study of 1,011 genomes by Peter et al. (4). Our pipeline recovered only 85.45 ± 2.84% of the models in that study (Fig. 4c). Further DIAMOND blastp search analyses revealed that 65.72 ± 6.02% of those models missed by our pipeline had the best hit to the “dubious” gene models as classified in the SGD database (S288c, version R64-2-1_20150113) (Fig. 4d), implying that most of those models missed by our pipeline are dubious models.

### Gene families

**Fig 5:**
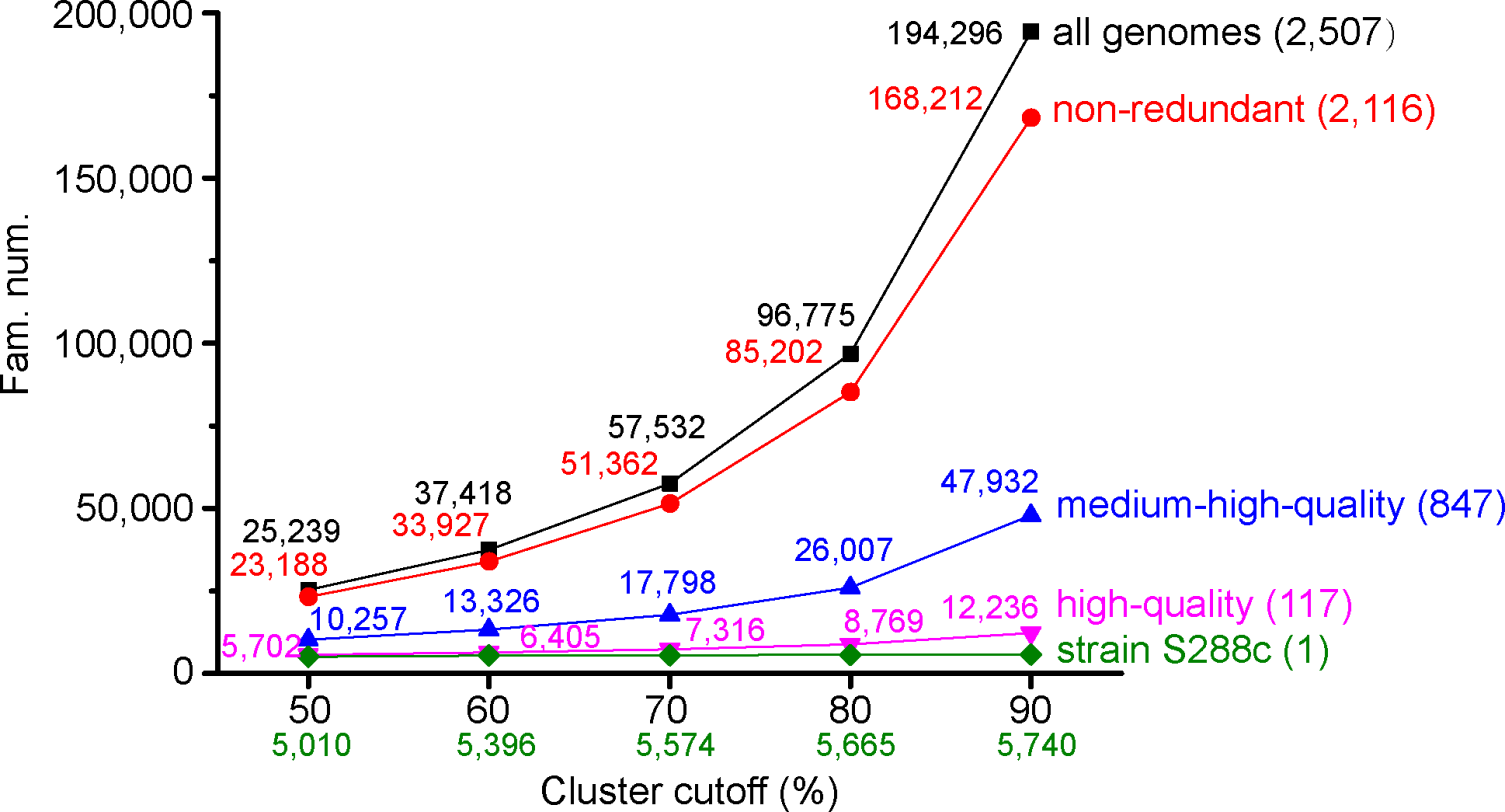
Gene families obtained based on different cluster cutoffs. Five cluster cutoffs, 50%, 60%, 70%, 80%, and 90%, were used. Four sets of genomes and the strain S288c genome were analyzed. All genomes, black; non-redundant, red; medium-high-quality, blue; high-quality, magenta; strain S288c, dark green. The number of genomes for each set was indicated in parentheses.

Gene families were clustered using a custom pipeline with different cutoffs (50%, 60%, 70%, 80%, and 90% sequence identity and coverage cutoff), and the resulting *S. cerevisiae* families (SCF) were referred to as SCF50, SCF60, SCF70, SCF80, and SCF90, respectively (19, 20). It was clearly shown that different cluster cutoffs resulted in different numbers of families. For all genomes (Fig. 5, black squares), the number of SCF90 is 7.7 times bigger than that of SCF50 (194,296 versus 25,239) [references (19, 20)]. In comparison, the 6,002 genes of the S288c reference assembly (accession GCF_000146045.2) were distributed in 5,010–5,740 families. Another three subsets of genomes (non-redundant, medium-high-quality, and high-quality) were picked (see below for details) and, similarly, cutoff selection greatly affected the number of the obtained families (Fig. 5).

**Fig 6:**
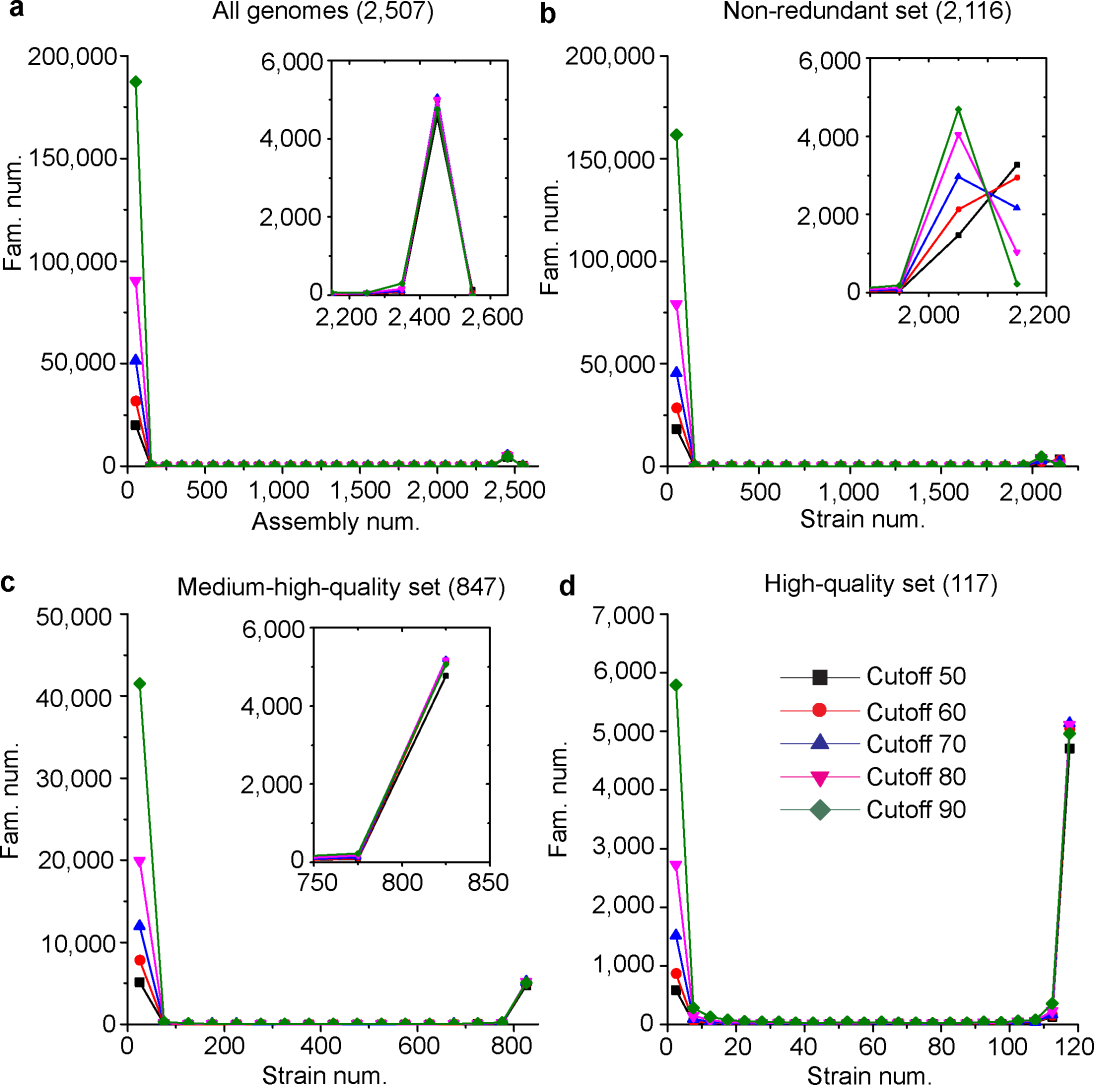
Distribution of gene families in different sets of genome assemblies. (a) All 2,507 assemblies. (b) The non-redundant set, including 2,116 strains. (c) The medium-high-quality set, including 847 strains. (d) The high-quality set, including 117 strains.

The distribution of the gene families among genome assemblies were analyzed (Fig. 6). It was shown that most families were narrowly distributed. For example, at the 50% cutoff, up to 20,074 families (79.5% of the total 25,239 families) were distributed in no more than 100 assemblies (4% of the total 2,507 assemblies). Among them, 14,303 families (56.7%) contained singleton genes found only in a single assembly. No families were present in all assemblies, which was consistent with the above observations that a few assemblies had too small sizes and thus too small number of genes (Fig. 3a). Furthermore, it was shown that the number of narrowly distributed families was much larger than that of widely distributed families and that the cutoff selection had a much higher impact on these narrowly distributed families than those widely distributed families (Fig. 6a). Analyses of the three subsets of genomes revealed the same trend (Fig. 6b through d).

### Marker gene set and evaluation of genome completeness

The above analyses had revealed a number of genomes with low qualities. To further evaluate the qualities of each assembly, a marker gene set was compiled specifically for *S. cerevisiae* (19, 20). A subset of 117 assemblies (referred to as the high-quality set) were firstly selected (Table S1), which contained 117 non-redundant strains with genome sequences of high continuity (contig number <100 and contig N50 >500 kb) and high similarity to the reference S288c genome (evaluated by maker-model ratio <33.33%). Gene families for this high-quality set were extracted from the five family series, SCF50, SCF60, SCF70, SCF80, and SCF90, respectively. A subset of 1,506 families shared by all the five family series and meeting a series of criteria were included in the marker gene set (see Materials and Methods for details).

**Fig 7:**
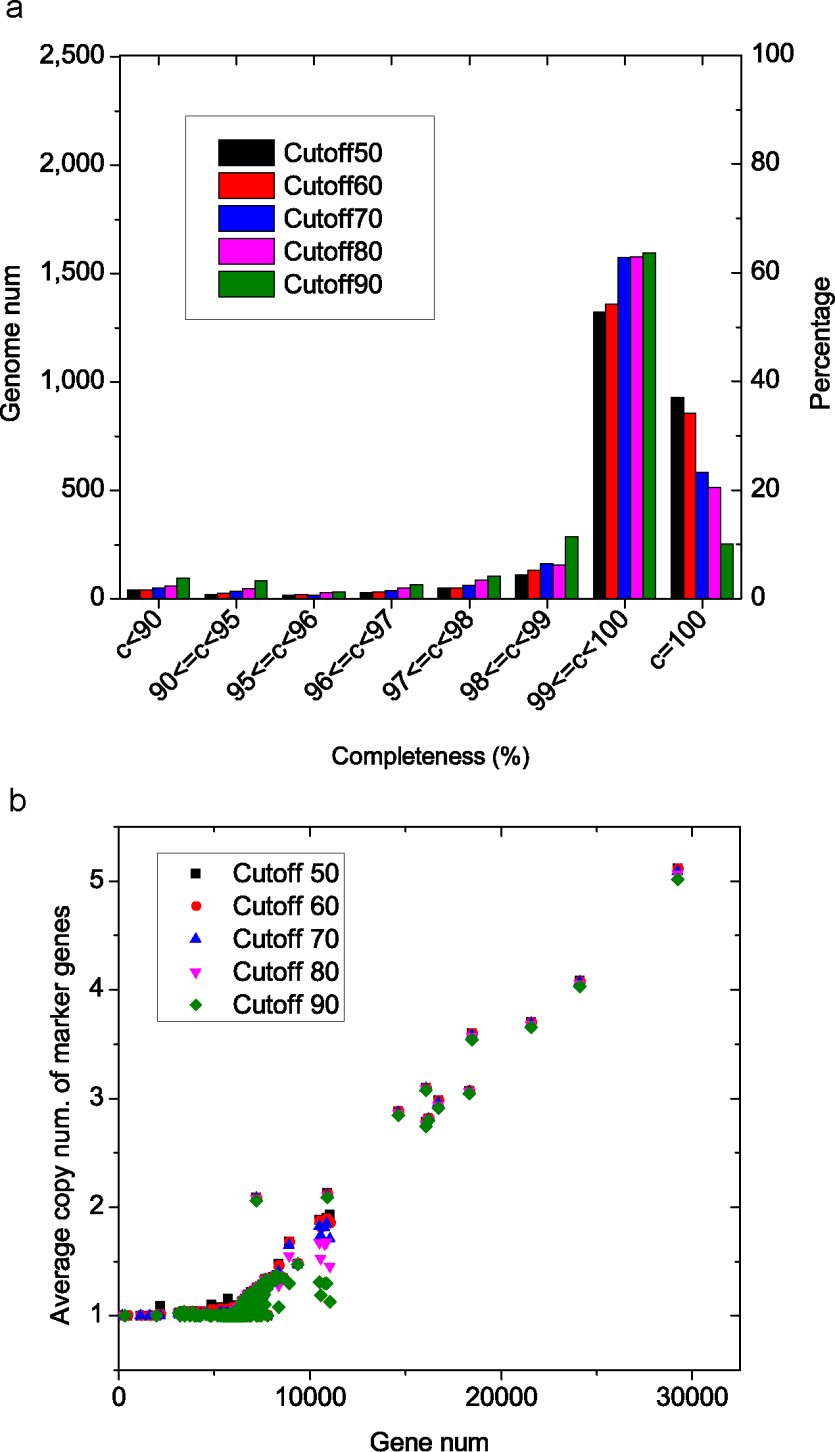
Evaluation of the genome completeness based on the marker gene set. (a) The distribution of completeness for all assemblies. (b) The relationship between the average homolog number in the marker gene families and the total gene number in the genome.

We used the obtained marker gene set to evaluate the completeness of all assemblies (6, 7) (Fig. 7a). Completeness was estimated based on different cutoffs (i.e., family series). As expected, more stringent cutoffs resulted in lower completeness values and ratios (Fig. 7a). With a medium cutoff of 70%, 581 assemblies (23.2% of the total) had a completeness of 100%, and 1,573 assemblies (62.7%) had completeness between 99% and 100%. A total of 80 assemblies (3.2%) had a completeness of <95%. With the stringent cutoff 90%, only 253 assemblies (10.1%) were complete and 1,595 assemblies (63.6%) had a completeness between 99% and 100%. Up to 177 assemblies (7.1%) had a completeness of <95%.

We also counted the homolog number in the marker gene family in each genome assembly (6, 7) (Fig. 7b). Analyses revealed 973 (SCF50) to 1,549 (SCF90) assemblies with one homolog per (marker gene) family (6, 7), and for other assemblies, more than one homolog was found (6, 7). Generally, a good correlation between homolog number and the total gene number was observed for large genomes (with >6,000 genes) (Fig. 7b). Our analyses found high average homolog numbers of marker genes for very large genomes, consistent with the reported diploidy and polyploidy of these genomes (8, 9).

### Possible low base qualities in a few assemblies

Besides continuity (e.g., contig number and N50), base quality is another parameter key to evaluate the genome quality. However, this information is missing from most databases. Here, we estimated the overall base quality of a genome by comparing the amino acid sequences of the marker genes. The assumption was simple. The marker gene sequences from the high-quality set of assemblies were thought to be of high quality and the sequence identities between the high-quality sequences and the homologous S288c sequences were used as the control. Sequences from other assemblies were compared with the S288c sequences and the obtained sequence identities were compared with the sequence identities for the high-quality set. Too low identities suggested possible low base qualities. Such analysis was performed for each assembly (6, 7).

**Fig 8:**
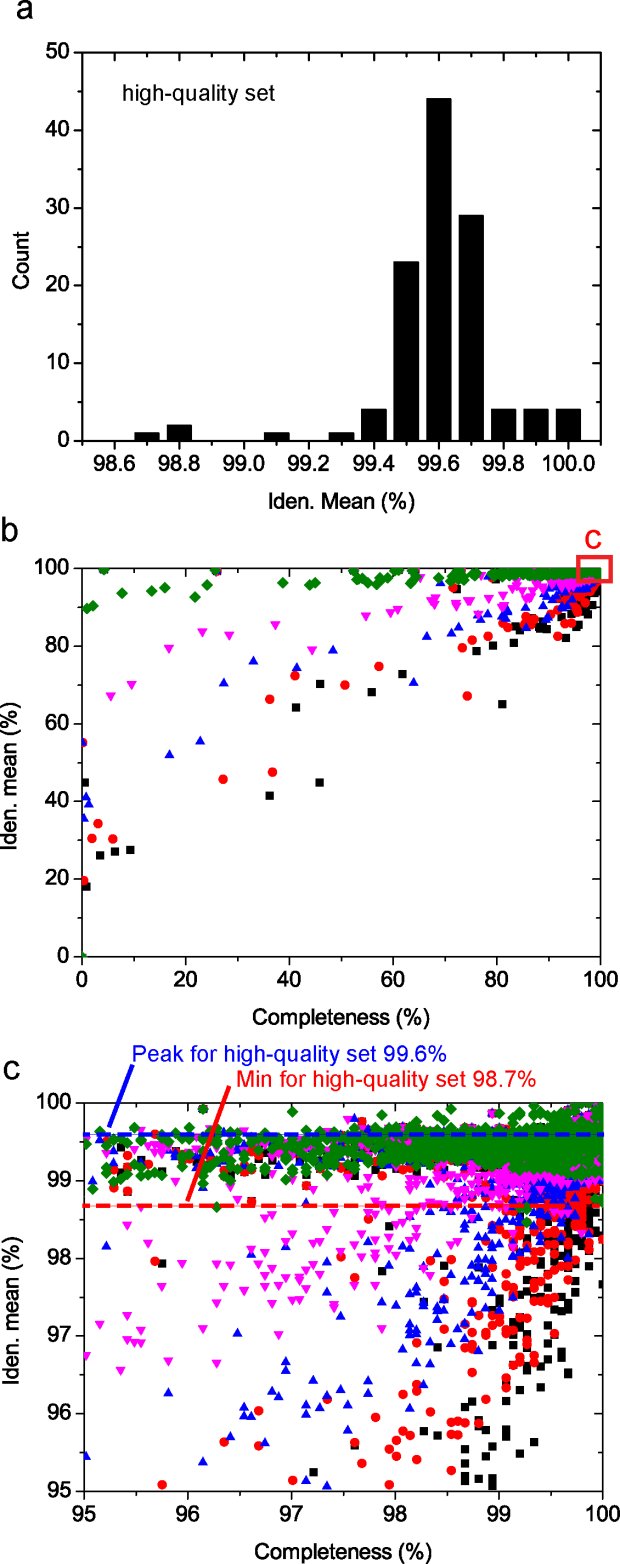
Evaluation of the base quality of the genomes. (a) Sequence identities of the marker genes between the high-quality set of genomes and the reference S288c. (b and c) The relationship between the marker gene sequence identities and the genome completeness for all assemblies except the high-quality set. For the marker gene family containing multiple homologs from one assembly, the one with the highest identity was analyzed.

The high-quality set of assemblies showed high similarities to the reference S288c, with the minimum identity of 98.72% and peak at 99.6% (Fig. 8a). Since the marker gene families were shared by all five family series (cutoffs), evaluation based on different family series resulted in the same similarity values for the high-quality set of genomes. For non-high-quality genomes, different similarities were obtained when different family series were used. Stringent cutoffs resulted in higher similarity values and, meanwhile, lower completeness values (Fig. 8b and c). Regardless of the cutoff used, genomes with low similarities to the reference had low genome completeness (Fig. 8b and c). Based on the stringent cutoff 90%, 82 assemblies (3.3% of the total 2,507 assemblies) had identities lower than the minimal identity (98.72%) of the high-quality set (Fig. 8b and c) and 520 assemblies (20.7% of total) had identities lower than 99.39% (5% of high-quality genomes had identities lower than this value). Based on the loose cutoff 50%, 224 assemblies (8.9% of total) had identities lower than the minimal identity (98.72%) of the high-quality set and 1,205 assemblies (48.1% of total) had identities lower than 99.39%. These results indicated that a large number of assemblies had low base qualities.

### Subsets of genomes and gene families

The above analyses revealed different sequence qualities of the assemblies analyzed in this study. To facilitate the reuse of the genome and gene sequences, we produced different subsets of genomes and genes. The first subset was the high-quality subset mentioned above, which was used to produce the marker genes. The second subset was the non-redundant subset (2,116 in number, 84.4% of total) (6, 7), which was generated by picking one high-quality genome assembly for each strain. The third subset was the medium-high-quality set (847 in number, 33.8% of total) (6, 7), which was picked from the non-redundant set and included strains with relatively high base qualities (identity to the reference ≥98.72%, i.e., the minimum for the high-quality set) and relatively high continuity (contig number <600) and completeness (>96%). Gene families were also generated for these three subsets of genomes by extracting genes from the corresponding genomes at different cutoffs (19, 20) (Fig. 5 and 6b through d).

### Pangenomic analyses

**Fig 9:**
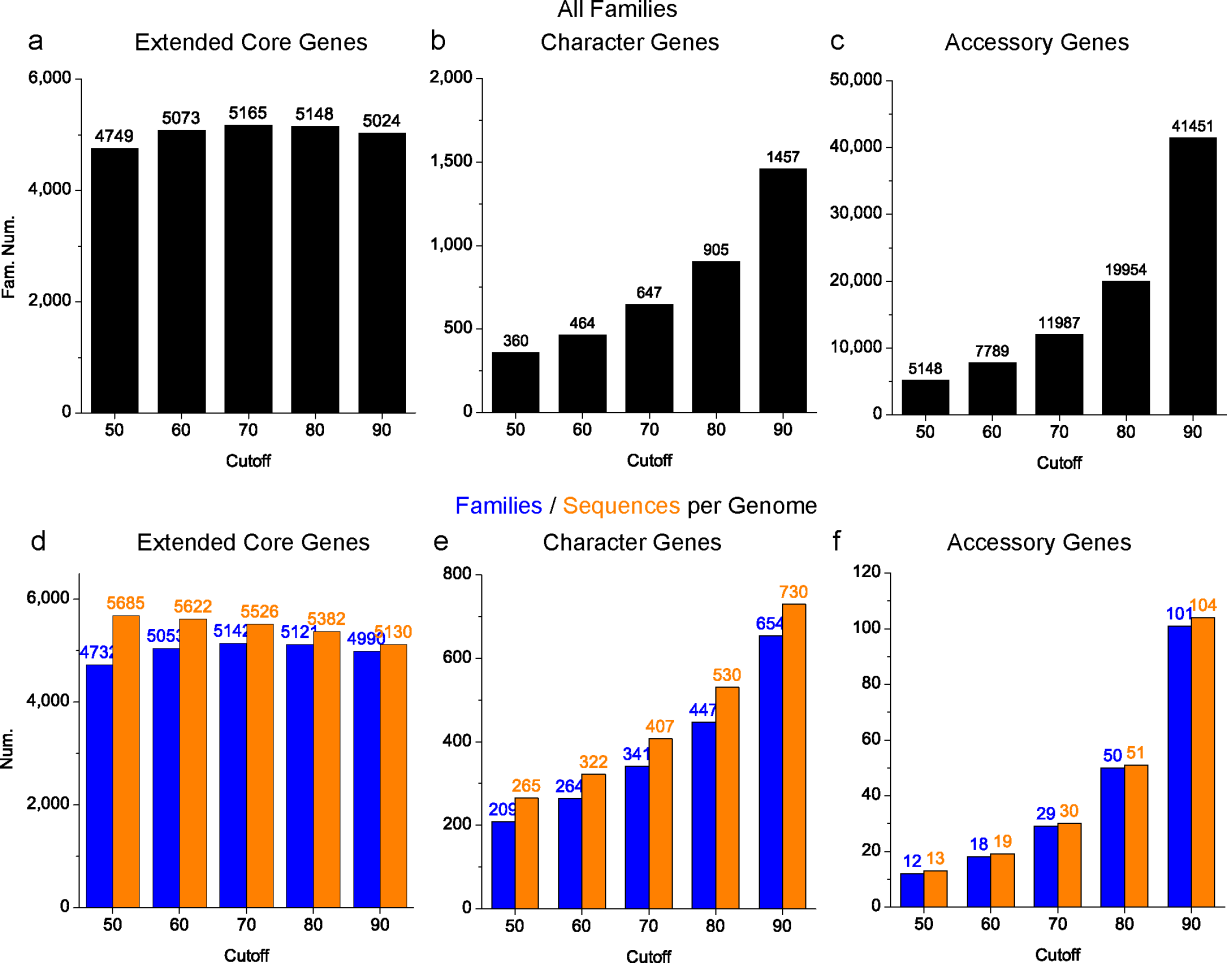
Pangenomic analyses based on the medium-high-quality set. (a–c) The total number of extended core gene families (a), character gene families (b), and accessory gene families (c) found in the medium-high-quality set. (d–f) The average number of gene families (blue) and sequences (orange) for extended core genes (d), character genes (e), and accessory genes (f) found in each medium-high-quality genome.

To alleviate the potential bias induced by the low-quality genomes, pangenomic analyses were performed based on the medium-high-quality subset (847 genomes). On average, each genome in this set contained 5,963 genes. Following previous studies (21), genes were classified into three categories: extended core genes (present in ≥95% of genomes), accessory genes (present in ≤5% of genomes), and character genes (in 5%–95% of genomes). It was shown that the number of extended core genes was relatively stable under different cutoffs (Fig. 9a). Differently, the numbers of character genes and accessory genes dramatically increased as the cutoff increased (Fig. 9b and c). These results were consistent with the above results that the number of narrowly distributed families was greatly affected by the cutoff selection and that of widely distributed families was less effected. The largest number (5,165) of extended core gene families was obtained at the medium cutoff 70%. The numbers of extended core genes, character genes, and accessory genes were counted for each genome (Fig. 9d through f), and these numbers were affected by the cutoff selection in the same way as the total number of gene families. At the medium cutoff 70%, each genome contained an average of 5,526 extended core genes (92.7% of the total) from 5,142 families, 407 character genes (6.8%) from 341 families, and 30 accessory genes (0.5%) from 29 families.

## DISCUSSION

A huge number of *S. cerevisiae* genome assemblies are available at the public domain. However, these genome assemblies are distributed at different sites and the genome annotation information is unavailable for a number of assemblies. Here, we compiled a genome set of 2,507 assemblies and (re-)annotated 2,506 of them using a uniform pipeline, providing a united resource for the community and facilitating the study of *S. cerevisiae* biology. Large-scale reanalyses of public available genome sequences had provided novel insights into the genomic diversity of *S. cerevisiae* (22). Several studies have annotated a large number of genomes, which revealed different numbers of protein-coding genes in the *S. cerevisiae* genome. McCarthy and Fitzpatrick (23) annotated genomes of 100 strains and revealed an average gene number of 5,759 in each genome. Li et al. (5) annotated 1,364 genomes and found that the *S. cerevisiae* genome contained 6,129 genes. Our analyses based on the 847 medium-high-quality genomes revealed an average gene number of 5,963 genes. This number was bigger than that (5,759) reported by McCarthy and Fitzpatrick (23) and smaller than that (6,129) reported Li et al. (5). Such differences may result from different annotation pipelines and different genome sets. Our pipeline was benchmarked using the reference S288c genome, and the results showed that our pipeline achieved a high recall rate and precision. Besides, different studies used different versions of annotations of the S288c genome as the reference. Li et al. (5) used the S288c genome from the SGD database as the reference, which contained many pseudogenes, dubious genes, and fragmented genes. Here, the S288c reference genome was downloaded from the NCBI RefSeq database (accession GCF_000146045.2), which contained fewer such genes, and the pseudogenes contained in the reference genome were excluded before being used as the reference in the annotation pipeline.

Genome continuity (e.g., N50) and presence ratio of marker genes are widely used in the evaluation of genome completeness. However, few metrics could be used to evaluate the base quality. The base quality evaluation is essentially different from the metric for genome completeness because the former focuses on the genes available in the current assembly while the latter focuses on the genes missing from the current assembly. Genome sequences available at the public database represent invaluable resources for the future study. However, the base qualities of these genome sequences should be carefully evaluated before being reused in analyses, or else, the low base qualities of some assemblies might lead to error in the conclusion for studies which rely heavily on base quality, e.g., phylogenetic analyses, gene conservation, and key residues in a protein. Here, a method for calculating the sequence similarity to the reference genome was developed to evaluate the base quality. Our analyses showed that the low sequence similarity was highly correlated to the genome completeness, strongly suggesting that the obtained low similarity is a signature of low sequence quality rather than the long phylogenetic distance and that such low sequence similarity resulted from the sequence assembly process that meanwhile resulted in the low completeness. To the authors’ knowledge, this study represents the first large-scale evaluation of base qualities of *S. cerevisiae* genomes available in the public domains. In this study, a large number of genomes were found to be of low base quality, and therefore, these genomes should be carefully evaluated before reuse in the future analyses.

Universal markers genes have been used in evaluating the genome completeness. BUSCO is a widely used marker gene sets for different lineages of eukaryotes and prokaryotes (24). Though the BUSCO eukaryotic data sets could be used to evaluate the genome completeness of *S. cerevisiae*, such data sets were compiled on both *S. cerevisiae* and non-*S*. *cerevisiae* organisms. Furthermore, these data sets included not only orthologs present in all studied organisms but also those present in >90% organisms studied. Here, a marker gene set was compiled specifically for *S. cerevisiae*, which was based only on high-quality strains of this species and contained only long and highly conserved, single-copy genes present in all strains. Thus, this marker gene set is expected to provide higher precision for the completeness evaluation of this species.

Different numbers of gene families have been found in different studies, including 7,796 families from 1,011 isolates in the Peter et al. study (4), 7,078 from 1,364 isolates in the Li et al. study (5), 7,750 from 100 isolates in the study of McCarthy and Fitzpatrick (23), and 7,888 from 368 isolates in the study of Han et al. (25). Many factors may contribute to the difference, including different sets of genomes, different gene prediction pipelines, and different clustering methods. While most studies reported gene families constructed based on one set of parameters (cutoff), it was shown that the number of gene families was affected by the cutoff selection (5). Here, we constructed gene families based on different cutoffs and obtained different numbers of gene families with different cutoffs (Fig. 6). Generally, this study revealed much higher number of gene families than those reported before. For example, with a very loose cutoff (coverage 50% and identity 50%), the selected subset of 847 medium-high-quality genomes contained 10,257 gene families, remarkably bigger than all previously reported numbers. With a stringent cutoff (coverage 50% and identity 50%), this number was up to 47,932.

**Fig 10:**
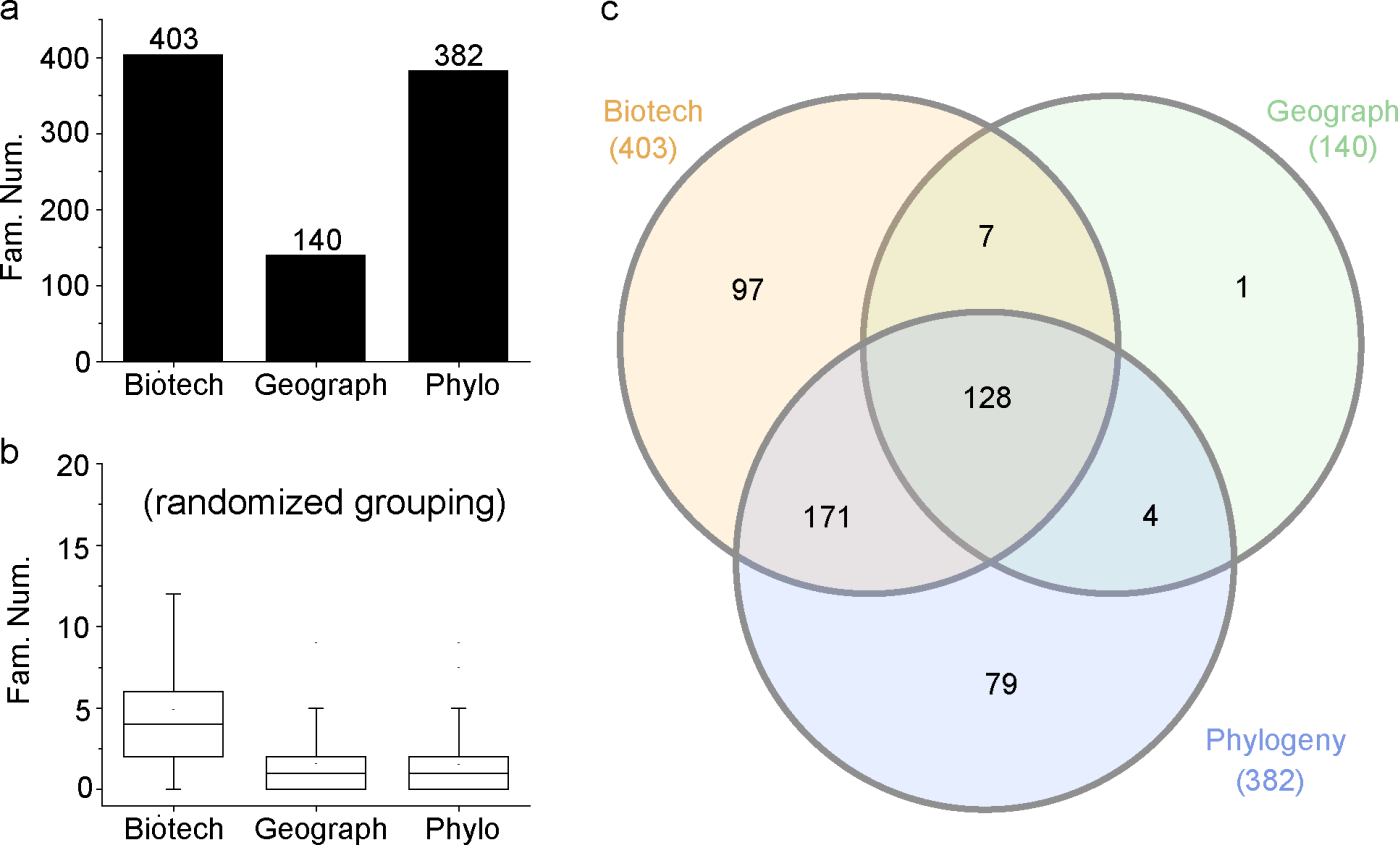
Gene families related to the environments/biotechnological potentials, geographic locations, and phylogenetic positions of strains. (a) The numbers of gene families recovered by different grouping schemes. (b) The numbers of gene families obtained by randomized grouping. (c) Venn diagram of gene families obtained by different schemes.

*S. cerevisiae* strains are widely distributed in different environments and used in bakery, brewing, distillery, and bioethanol production (2). They are also regarded as an emerging fungal pathogen (26). It is expected that the gene content variations among strains are related to the environmental adaptation or biotechnological potentials. By classifying the strains into groups based on their sources (environments/biotechnological applications, geographic locations, and phylogenetic positions, respectively), we found a number of gene families showing remarkable inter-group differences in the gene number (Fig. 10a, see Materials and Methods for details). In comparison, randomized grouping produced much lower number of such families (Fig. 10b), suggesting the families found above are functionally relevant. Interestingly, a large number of detected families were shared by two or three grouping schemes (Fig. 10c), suggesting that the variation of homolog number in these families could be explained by more than one scheme. It was also noted that there were 97 families that were specific to the environment/biotechnological potential grouping. For example, these included a guanosine diphosphate (GDP)-mannose transporter family (SCF70_00667, depleted in the milk group), a purine-cytosine permease family (SCF70_00689, depleted in the milk group), an alpha-glucoside permease family (SCF70_01366, depleted in the milk group), an oligopeptide transporter (SCF70_01850, enriched in the milk group), and a putative glycosylphosphatidylinositol (GPI)-anchored mannoprotein family (SCF70_04037, depleted in the bioethanol and palm wine groups).

This study provided one complete annotated genome data set of *S. cerevisiae*, an important workhorse for fundamental biology, biotechnology, and industry. We plan to update this genome data set periodically to incorporate newly sequenced *S. cerevisiae* genomes.

## MATERIALS AND METHODS

### Genome assemblies

All genome assemblies (1,496) under the name “*Saccharomyces cerevisiae*” available at the NCBI genome database at the time of this study (December 2023) were obtained. All genome assemblies (1,011) used in the Peter et al. study (4) were obtained. To simplify the analysis, the genome assemblies were renamed using a four-letter tag, with those from NCBI being assigned a four-letter tag beginning with “A” and those from the Peter et al. (4) being assigned a four-letter tag containing the original three-letter tag plus a leading letter “B.” The assembly GCF_000146045.2 of the reference strain S288c was used as the reference in the following analyses and assigned a tag “S288C.”

### Annotation

A two-step pipeline was used to predict protein-coding genes for all assemblies except S288c, which was used as one of the reference genomes. Firstly, the gene models were predicted with the help of BLASTN version 2.11.0+ (https://ftp.ncbi.nlm.nih.gov/blast/executables/blast+/). Each genome was searched against the S288c genome with BLASTN. All S288c gene models in each aligned segment of the S288c genome were checked and the complete models (i.e., with both the start codon and stop codon) were copied to the query genome if there were no indels in the alignment of the model. The obtained candidate models were then inspected for the start codon and stop codon. If a non-stop codon was found at the stop codon position of the model, the model was extended downward (within 300 bp) to the first stop codon encountered. If a non-start codon was found at the start codon position of the model, the upstream sequence (within 300 bp) was scanned to find the first possible start codon. If no start codon was found in the upstream sequence before meeting a stop codon, the downstream sequence (within 300 bp) of the original start codon was scanned to find the first start codon. If no start codon or stop codon was found, the model was discarded. The obtained model was also checked to find whether there was a stop codon in the middle of the model and if there was, the model was regarded to be a pseudogene and discarded. The protein sequences were generated based on the standard genetic code (NCBI transl_table = 1), unless otherwise the gene was aligned to the S288c mitochondrial DNA sequence, in which case the yeast mitochondrial code (NCBI transl_table = 3) was used. The obtained gene models were referred to as blastn-models. This step was used to generate models for genes high similar to their S288c homologs (no indels in the coding regions).

Next, the genome regions without blastn-models were scanned for genes using the MAKER program version 3.01.03, an annotation pipeline which integrates support for multiple homology-based and *ab initio* gene prediction tools and produces accurate annotations (27). In this study, homology-based gene predictions were produced by BLASTN version 2.2.28+, BLASTX version 2.2.28+, and EXONERATE version 2.2.0 (28) and *ab initio* gene predictions were produced by AUGUSTUS version 3.4.0 (29). The mRNA sequences of S288c gene models (GCF_000146045.2_R64_rna.fna) were used as the reference for the gene predictions based on BLASTN. Protein sequences of *S. cerevisiae* S288c and another five *Saccharomyces* species (*Saccharomyces arboricolus*, GCA_000292725.1; *Saccharomyces eubayanus*, GCA_001298625.1; *Saccharomyces kudriavzevii*, GCA_000167075.2; *Saccharomyces mikatae*, GCA_000167055.1; *Saccharomyces paradoxus*, GCF_002079055.1) were used as the reference for the predictions based on BLASTX and EXONERATE. To reduce the size of the reference sequence database, those non-S228c proteins highly similar to their S288c homologs (identity ≥70%, query coverage ≥95%, subject coverage ≥95%) were excluded from the database. As a result, 9,910 protein sequences were included in the database. The AUGUSTUS gene prediction species model was “saccharomyces.” The obtained models were checked to remove those of too short length (<15 aa) and the resultant models were referred to as the maker-models. The blastn-models and maker-models were pooled to obtain the final models.

### Homologous gene families

To reduce the usage of computation resources, the obtained gene models were clustered into families with a two-step procedure. The first step was to create initial families. The reference S288c gene models were clustered into gene families based on the BLASTP (version 2.11.0+) identity cutoff and alignment coverage cutoff by using the single linkage clustering algorithm. To simplify the analysis, the identity cutoff value and the coverage cutoff value were the same and were mentioned as cluster cutoff. Then, each blastn-model was assigned to the gene family to which their reference S288c model belongs. Next, the maker-models were searched against the reference S288c models. The gene with an identity > cluster cutoff and alignment coverage > cluster cutoff (to a reference S288c model) was classified to the family to which the reference S288c gene belongs. Then, the remaining maker-models were clustered into families based on the single linkage clustering algorithm with the same cluster cutoff. All obtained families were pooled to get the initial families.

The second step was to merge the initial families that were closely related (but not detected in the first step) into the same final families. To achieve this goal, the longest sequence in each initial family was chosen as the representative sequence and all-versus-all sequence searches were then performed based on the representative sequences with DIAMOND (version 2.0.15.153) to find the related family pairs (30). A pair of initial families were chosen for further all-versus-all inter-family DIAMOND sequence search if the similarity of the representative sequences met the following criteria: match coverage > coverage cutoff – 10 and match identity > identity cutoff – 10. If there were matches meeting the coverage cutoff and identity cutoff, these two initial families were marked as related family pair. The related initial family pairs were then clustered by using the single linkage clustering algorithm. The obtained clusters were the final gene families.

In this study, five cluster cutoff values were used: 50%, 60%, 70%, 80% and 90%, and the obtained SCF were referred to as SCF50, SCF60, SCF70, SCF80, and SCF90, respectively.

### Marker gene selection and genome quality evaluation

For each of the five family series for the 117 high-quality assemblies, an initial set of marker genes were selected based on the following criteria: (i) present all genomes, (ii) with only one homolog per genome, (iii) highly conserved (minimal pairwise global identity ≥90%) within family, (iv) with low ratios of gaps in the multiple sequence alignment of the family (length of alignment / length of reference sequence from S288c ≤ 1.05), and (v) with a relative long sequence (≥200 aa) for the representative sequence in S288c. The resultant five initial sets were compared and those families shared by all five sets were obtained as the final marker gene set.

The genome completeness was evaluated by counting the number of marker genes present in the genome. To obtain the sequence similarity of a genome with the reference strain S288c, all sequences of each marker gene family were firstly aligned using MAFFT version 7.520 with default parameters (31). Then, for each family, the sequence identities with the corresponding S288c homolog were calculated based on the alignment. When a genome contained more than one sequence belonging to the same gene family, only the sequence with the highest identity to the S288c homolog was selected to represent the genome. Finally, the mean identity of all marker gene families was used to represent the sequence identity of the genome (assembly) to the reference S288c.

### Comparison of homolog numbers between different groups of strains

All the following analyses were based on the medium-high-quality subset of SCF70 families. Three schemes were used to produce predefined groups, including environments/biotechnological applications, geographical locations, and phylogenetic positions. For the environments/biotechnological applications, 12 groups were defined, including beer (35 strains), bioethanol (13), clinical (53), distillery (32), dough (56), fruit (40), milk (11), palm wine (12), plant (101), rice wine (29), soil (17), and wine (44). For geographical locations, five groups were defined, including Africa (43), East Asia (287), Europe (292), North America (73), and South America (28). For phylogenetic positions, six groups were defined, including sub1 (159), sub2 (174), sub3 (85), sub4 (90), sub5 (40), and sub6 (43).

For each set of predefined groups, the Kruskal-Wallis H-test was performed for each family by using the function scipy.stats.kruskal() in Python 3.11.4. The family with *P*-value <0.05 was further subject to Dunn’s test by using the function scikit_posthocs.posthoc_dunn() in Python 3.11.4. A pair of groups was considered significantly different if they had a *P*-value <0.05 and their difference in the average homolog number was greater than 0.5. A family with at least one such significantly different group pair was considered to be a family with inter-group difference.

To obtain the phylogenetic positions of the strains, the marker genes that were present in all medium-high-quality set of genomes were selected for multiple sequence alignment using MAFFT version 7.520 with default parameters. Then, the obtained individual alignments (401 in number) were concatenated and used for further phylogenetic reconstruction using FastTree version 2.1.11 with default parameters (32). The phylogenetic groups were obtained by manual selection of large subtrees.

### Data sets deposited at figshare and Zenodo

Detailed information for the 2,507 *Saccharomyces cerevisiae* genome assemblies studied are presented as a Microsoft Excel file (.xlsx) (6, 7). The genome sequences re-annotated were presented as separate files for each assembly (in the fasta format) (33, 34). The coordinate files of the predicted gene models are presented for each assembly (in the gff format) (10, 11). The nucleotide and amino acid sequences of the predicted gene models are presented for each assembly (in the fasta format) (12–15). The predicted protein functions were presented in text files for each assembly (16, 17). The protein families obtained with different cluster cutoffs for different sets of genome assemblies as well as the marker genes are presented in text files (19, 20). In each file, a row indicates a family and a column (separated by TAB) indicates a genome assembly. Protein IDs for multiple homologs from the same assembly are separated by “|.” The family ID is shown in the first column and the tag for each assembly is shown in the first row. Cluster cutoffs used are 50%, 60%, 70%, 80%, and 90%. Genome sets shown are all genomes, non-redundant genomes, medium-high-quality genomes, and high-quality genomes.

## Data Availability

The data sets presented here are deposited both at figshare (https://figshare.com/projects/Annotation_and_Comparative_Analyses_of_2032_Saccharomyces_cerevisiae_Genomes/140098) and at Zenodo (see details in References). Individual files are described below. Codes for genome annotation and gene family clustering have been deposited at GitHub.

## SUPPLEMENTAL MATERIAL

Table S1 spectrum.03582-23-s0001.xlsx Detailed information for the genome assemblies studied.

ASM does not own the copyrights to Supplemental Material that may be linked to, or accessed through, an article. The authors have granted ASM a non-exclusive, world-wide license to publish the Supplemental Material files. Please contact the corresponding author directly for reuse.
