# Erratum for Wang et al., “Annotation of 2,507 *Saccharomyces cerevisiae* genomes”

**DOI:** 10.1128/spectrum.02374-24

**Published:** 2024-11-11

**Authors:** Meng Wang, Xuan Li, Xian Liu, Xiaoping Hou, Yang He, Jun-Hong Yu, Shumin Hu, Hua Yin, Bin-Bin Xie

**Affiliations:** 1 State Key Laboratory of Biological Fermentation Engineering of Beer, Tsingtao Brewery Co., Ltd, Qingdao, China; 2 Microbial Technology Institute and State Key Laboratory of Microbial Technology, Shandong University, Qingdao, China

## Erratum

Volume 12, no.4, e03582-23, 2024 https://doi.org/10.1128/spectrum.03582-23. The affiliation line and the affiliation numbers for each author should appear as shown in this erratum.

